# Distribution of sources of household air pollution: a cross-sectional study in Cameroon

**DOI:** 10.1186/s12889-021-10350-6

**Published:** 2021-02-08

**Authors:** Miranda Baame Esong, André Pascal Goura, Bertrand Hugo Ngahane Mbatchou, Berenice Walage, Herman Styve Yomi Simo, Romarique Mboumo Medjou, Martial Pianta Sonkoue, Cyrielle Douanla Djouda, Rose Suzie Fowoh Ngnewa, Milaine Sandra Teugueu Guiagain, Brice-Donald Kemnang Agokeng, Olivia Tania Megaptche Homla, Dan Pope, Jerome Ateudjieu

**Affiliations:** 1 National institute of Human Research (HIHR) CLEAN-Air (Africa) Global Health Research Group (GHRG), Mbalmayo, Cameroon; 2 Meilleur Accès aux Soins de Santé (M.A. SANTE), Yaoundé, Cameroon; 3 Douala General Hospital, Douala, Cameroon; 4 Faculty of Medicine and Pharmaceutical Sciences, Department of Public Health, University of Dschang, Dschang, Cameroon; 5 Department of Public Health and Policy, University of Liverpool, Liverpool, UK

**Keywords:** Indoor air pollution, Household air pollution, LPG, Fuel type, Dschang-Cameroon

## Abstract

**Background:**

Household air pollution (HAP) is a recognised risk factor for many diseases, including respiratory diseases, cardiovascular/circulatory disorders, adverse pregnancy outcomes and cataracts. Population exposure to biomass fuels, including wood, varies among countries and from one fuel source to the other. This study aimed to investigate the different sources of HAP in peri-urban and rural communities in Cameroon.

**Methods:**

A cross-sectional survey was conducted in a representative sample of households from the Dschang Health District (DHD) region. This included 848 homes in which a range of fuels for cooking including biomass (firewood, charcoal, sawdust), kerosene and liquefied petroleum gas (LPG) were used both indoors and outdoors.

**Results:**

Of the study households, 651 (77%) reported exclusive use of firewood and 141 (17%) reported using more than one source of fuel. Exclusive use of firewood was greater in rural communities (94%) than in peri-urban communities (38%). In peri-urban communities, use of multiple fuels including LPG, wood, sawdust and kerosene, was more common (44.75%). A total of 25.03% of households in both peri-urban and rural communities reported using bottled gas (or liquified petroleum gas (LPG) for cooking. Motivations for choice of fuel included, affordability, availability, rapidity, and cultural factors.

**Conclusion:**

Wood is the main cooking fuel in both peri-urban and rural communities in the Dschang Health District. Supporting households (especially those with limited resources) to adopt LPG equipment for cooking, and use in a more exclusive way is required to help reduce household air pollution.

## Background

Over 3 billion people rely on biomass fuel (BMF) as their main source of domestic energy [1, 2]. BMF, including wood, charcoal, dung and crop residue, accounts for as much as 95% of fuel usage in lower income countries [3, 4]. Studies have shown that there is an increase in the risk of respiratory morbidity and chronic obstructive pulmonary diseases among individuals using biomass fuels [1, 5, 6].

The World Health Statistics estimated in 2018 that acute lower respiratory infection (ALRI) is one of the leading causes of child mortality in the world, accounting for up to 15% of fatalities among children under five, almost all of them in developing countries [7]. The most recent demographic health survey for Cameroon (2018) presented 1% of children under five had symptoms of acute respiratory infections in the two weeks before the survey [8]. A recent study carried out in Bamenda regional hospital showed a prevalence of 54.7% acute respiratory infections amongst infants less than five years [9]. Household air pollution (HAP) is thought to cause about one-third of ARI cases [1]. This makes solid fuels the second most important environmental cause of disease [6, 7] and the fourth most important cause of overall excess mortality in developing countries [6]. In addition to impact on mortality, HAP may have long lasting effects on general health and well-being: early exposure to HAP during childhood may stifle lung development, suggesting that the cost of this pollution may continue later in life. In fact, a growing literature indicates that environmental insults at early ages can have long lasting influences on human health and productivity [10].

According to the World Health Organization (WHO) reports, deaths estimated to be related to ambient air pollution globally tripled, from 1.3 million in 2008 to 3.8 million in 2016 [11]. More than two million premature deaths each year were related to air pollution. Globally, seven million deaths were attributable to the joint effects of household and ambient air pollution in 2016 [1, 11].

In Cameroon, the industrial sector is still developing, so ambient air pollution has not reached health-damaging levels, yet HAP is known to have caused an estimated 11,400 premature deaths in Cameroon [12]. One of the highest contributors to ambient air pollution in Cameroon is therefore BMF for cooking and space heating. To reduce this burden in the LMIC, the World Health Organisation (WHO) recommended the adoption and scaling up of clean fuels [13, 14]. The real-time reliable knowledge about the implementation of this recommendation and the relevant needs is very still disparate in Cameroon according to areas of the country and time covered by published data meanwhile Cameroon is a multicultural country with more [15–17] than 280 different tribes and traditions.

Only few studies conducted in Northern and Southern Cameroon, have been published so far. These studies have revealed that firewood, kerosene and liquefied petroleum gas (LPG) were the main sources of cooking energy in households with close to 90% of households rely on solid fuel in rural areas [17–20]. Prices of fuels, socioeconomic status, household wealth, were listed as some of the determinants of LPG adoption in households. This study was designed to highlight the various cooking fuel types (sources of HAP) in another region of Cameroon to provide real-life information that can guide specific interventions.

## Methods

This was a cross-sectional community-based study. A pretested questionnaire was administered to heads of households or representatives in randomly selected urban and rural households in the Dschang Health District (DHD) in Cameroon from March to July 2018 to estimate the distribution of sources of household air pollution and characteristics. The DHD is a cosmopolitan district with an estimated population of 221,037 inhabitants in 2018. This district was chosen because of the diversity and big size of its population and ability to compare rural and urban household characteristics. It is made up of 22 Health Areas (HA) classified into urban and rural.

The minimum sample size was estimated assuming a proportion of 50% of the population used biomass (since the prevalence of HAP was unknown, to the best of our knowledge), a 95% confidence level, a 5% relative precision, a cluster effect of 2 and a 20% nonresponse rate.

Multistage stratified random sampling was performed among 22 HA to select 11 HA with equal representation of urban (2/4) and rural (9/18) health areas. Within selected health areas, villages/quarters were selected by simple randomisation from the list of clusters obtained from the National Institute of Statistics. The sample required from each health area was calculated with respect to their representativity in the general population (2018). In villages/quarters, a guide was solicited from the local traditional authorities and the central spot was identified. On the left/right side of the street, one household was targeted after one was skipped till the end of the street. The process was repeated until the expected sample size of households for the village was reached.

A household was defined in this study as one or more persons living together, sharing the same roof and kitchen. All unoccupied buildings were excluded and replaced by the one immediately next to it.

### Data collection and management

In targeted households, data was collected from head of households or representative after verbal/signed consent was obtained. Data was collected using a structured questionnaire administered in face to face interview by a trained surveyor. The questionnaire developed by the study team was pretested in households of one of the DHD health areas which was not selected and included in the study. For each household, data was collected on socio demographic information and characteristics of indoor sources of air pollution including fuels used for cooking. Primary cooking fuel was defined as the fuel used mainly by a household for cooking [4]. Secondary cooking fuel was defined as the fuel used as a backup for fuel-specific cooking activities by a household.

Resulting forms were verified daily to assess quality and completion. The resulting database was cleaned and analyzed by a statistician using Epi info version 7.2.2 software. Main analysis performed were proportions with a 95% confidence interval and tables were designed using MS-Excel 2013.

### Ethical considerations

The proposal of this study was submitted and evaluated by the National Ethics Committee of Human Health Research of Cameroon and approval was given with the reference number **1030.** Prior to this evaluation, authorization was obtained in a signed document from local health authorities of the DHD. For each village/quarter, authorization was obtained from local traditional authorities. Data was collected from consenting households.

## Results

As detailed in Table 1, 11 health areas were selected – 2/4 in urban and 9/18 in rural setting - leading to a final sample of 848 households (98% response rate) within 85 villages/quarters. The study sample included 257 urban and 591 rural household. Approximately 80% of respondents were females. The mean age of respondents was 38 (SD: 18.8) years, median age was 33 [range: 15–97] years. Average household size was 5 (Table 2). Nearly 15% of female respondents had never been to school and 60% of female participants reported peasant farming as their main occupation.

**Table 1:** Distribution of households reached in targeted health areas with population in 2018 by cluster and setting

Targeted Health Areas of the study	Urban or Rural	Population size in 2018 (inhabitants)	Clusters (Reached/Expected)	Households reached
Fiala-Foreke (1)	Urban	34,760	20/20	197
Balevouni (2)	Rural	1856	1/1	12
Nkeuli (3)	Rural	2691	2/2	19
Fotetsa (4)	Rural	5128	3/3	28
Maka (5)	Urban	10,804	6/6	60
Fonakeukeu (6)	Rural	5149	3/3	30
Lepoh (7)	Rural	10,472	6/6	60
Ndoh-Djuttitsa (8)	Rural	13,663	9/9	93
Baleveng (9)	Rural	20,658	12/12	118
Doumbouo (10)	Rural	16,908	10/10	99
Mbeng (11)	Rural	20,508	13/13	132
**TOTAL**	**/**	**142,597**	**85/85**	**848**

**Table 2:** Socio-demographic presentation of the study sample

Characteristics	Modalities	Urban	Rural	Total	***P*** value
Reached households (*n*)	/	257	591	848	
Response rate (%)	/	99.2	100	99.8	
Gender [*n* (%)]	Female	187 (72.8)	481 (81.4)	668 (78.8)	0.005
	Male	70 (27.2)	110 (18.6)	180 (21.2)	
Age of respondent (μ ± SD)	/	31.1 ± 12.9	41.5 ± 20.0	38.3 ± 18.8	< 0.0005
Average household size (μ ± SD)	/	5.4 ± 2.8	4.7 ± 2.4	4.9 ± 2.6	< 0.0005
Mother’s level of education [*n* (%)]	No school	13 (5.5)	96 (16.3)	109 (13.2)	< 0.0005
	Primary	63 (26.7)	307 (52.0)	370 (44.8)	
	Secondary	112 (47.5)	181 (30.7)	293 (35.5)	
	Higher	48 (20.3)	6 (1.0)	54 (6.5)	
Father’s level of education [*n* (%)]	No school	10 (4.4)	103 (18.0)	113 (14.1)	< 0.0005
	Primary	70 (30.6)	275 (48.2)	345 (43.1)	
	Secondary	92 (40.2)	168 (29.4)	181 (32.5)	
	Higher	57 (24.9)	25 (4.4)	6 (10.2)	
Mother’s occupation [*n* (%)]	Housewife	47 (20.0)	29 (4.9)	76 (9.3)	< 0.0005
	Farming	55 (23.4)	452 (77.4)	507 (61.9)	
	Self-employment	69 (29.4)	73 (12.5)	142 (17.3)	
	Civil servant	29 (12.3)	23 (3.9)	52 (6.3)	
	Student	35 (14.9)	7 (1.2)	42 (5.1)	

### Main sources of household air pollution in the DHD

**Fig. 1:**
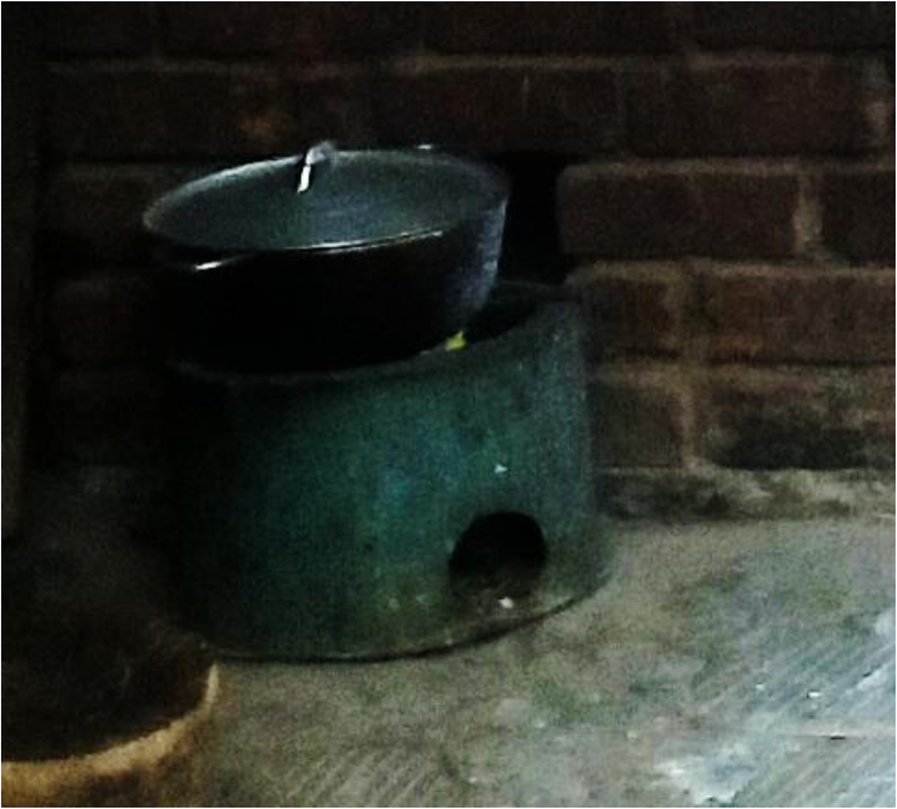
Picture of a charcoal fuel sources, taken in a household enrolled in the study

**Fig. 2:**
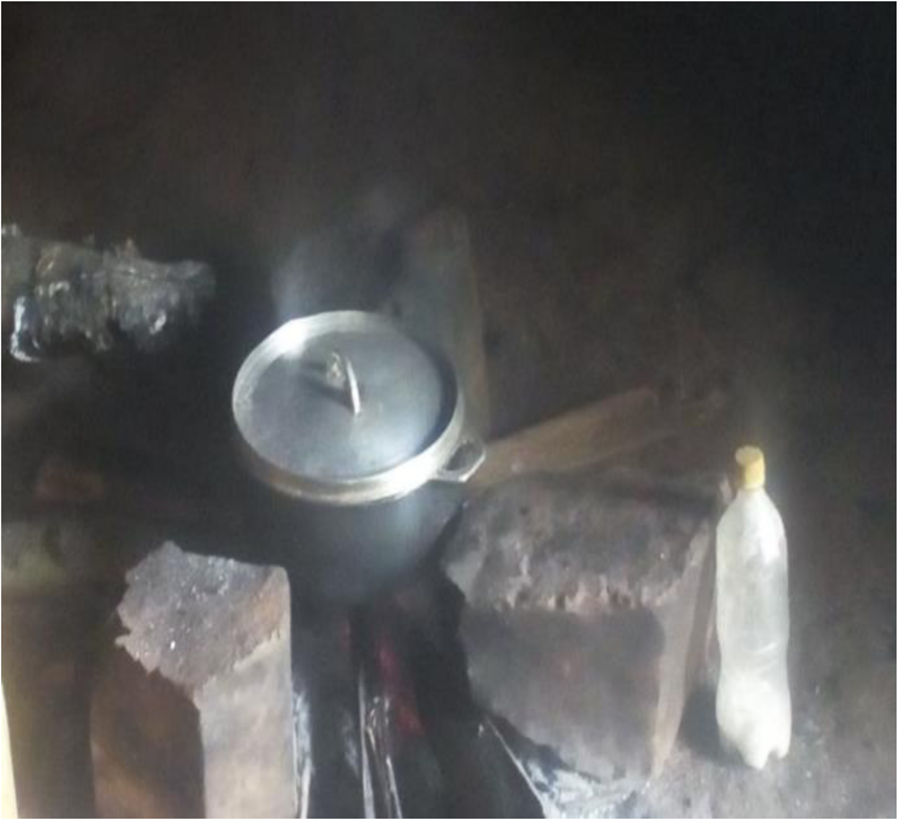
Picture of a firewood fuel source at household level. This picture was snapped in a household enrolled in the study

From this study, 90% of households used firewood and about 75% (630 households) of them exclusively used wood. Almost all households (98%) used wood to meet at least some of their cooking needs in rural HAs. Our results also reveal that 25% household have access to domestic gas for cooking, with a higher proportion in urban areas (55.6%) (Table 3). Figures 1 & 2 show the pictures of a charcoal and firewood fuel source, snapped in two separate households enrolled in the study. *P*-value less than 0.0005 as presented in Table 3 means the difference in proportion between urban and rural settings is statistically significant. It is important to note that no one was found to use an electric cooker or a micro wave.

**Table 3:** Distribution of cooking fuel types in the households of the Dschang Health District

Cooking instruments	Urban [***n*** (%)]	Rural [***n*** (%)]	Total [***n*** (%)]	***P*** value
Firewood only*	97 (37.7)	533 (74.3)	630 (74.3)	< 0.0005
Firewood	191 (74.3)	577 (97.8)	768 (90.6)	< 0.0005
Gas only*	38 (14.8)	10 (1.7)	48 (5.7)	< 0.0005
Gas	143 (55.6)	69 (11.7)	212 (25.0)	< 0.0005
Kerosene stove	14 (5.5)	12 (2.0)	26 (3.1)	0.008
Charcoal	33 (12.8)	6 (1.0)	39 (4.6)	< 0.0005
Saw dust	26 (10.1)	6 (1.0)	32 (3.0)	< 0.0005

* Exclusive use of the fuel type

Among the interviewed households, approximately 75% use more than one cooking fuel type (Table 4).

**Table 4:** Distribution of multiple cooking fuel type utilization in the Dschang Health District

Number of cooking fuel sources	Urban [***n*** (%)]	Rural [***n*** (%)]	Total [***n*** (%)]	***P*** value
More than one source of cooking fuel	114 (44.4)	67 (11.3)	181 (21.3)	< 0.0005
More than two sources of cooking fuel	29 (11.3)	4 (0.7)	33 (3.9)	< 0.0005
More than three sources of cooking fuel	8 (3.1)	0 (0.0)	8 (0.9)	< 0.0005

### Factors influencing the choice of cooking fuel type

Nearly 60% of households choose their fuel type based on affordability and only 18% based on availability (Tables 5 and 6). Availability meant the presence of the fuel type in the surroundings of the household.

**Table 5:** Distribution of reasons influencing choice of cooking fuel type in the Dschang Health District

Reasons	Urban [n (%)]	Rural [n (%)]	Total [n (%)]	***P*** value
Affordability	130 (50.6)	375 (63.4)	505 (59.5)	< 0.0005
Availability	27 (10.5)	122 (20.6)	149 (17.6)	
Rapidity	44 (17.1)	31 (5.2)	75 (8.8)	
Culture	8 (3.1)	40 (6.8)	48 (5.7)	
Easy to use	33 (12.8)	17 (2.9)	50 (5.9)	
Cleanliness	10 (3.9)	5 (0.8)	15 (1.8)	
Other reason	5 (1.9)	1 (0.2)	6 (0.7)

**Table 6:** Distribution of main reasons of choice per main cooking fuel types in the Dschang Health District

	Firewood [n (%)]				Gas [n (%)]				Charcoal [n (%)]			
**Reasons**	**U**	**R**	**T**	***P*** **value**	**U**	**R**	**T**	***P*** **value**	**U**	**R**	**T**	***P*** **value**
Affordability	118 (61.8)	373 (64.5)	491 (63.9)	< 0.0005	52 (36.4)	37 (53.6)	89 (42.0)	0.08	16 (48.5)	4 (66.7)	20 (51.3)	0.05
Culture	8 (4.2)	40 (6.9)	48 (6.2)		4 (2.8)	3 (4.4)	7 (3.3)		0 (0.0)	0 (0.0)	0 (0.0)	
Rapidity	22 (11.5)	28 (4.8)	50 (6.5)		36 (25.2)	9 (13.0)	45 (21.2)		5 (15.2)	0 (0.0)	5 (12.8)	
Cleanliness	5 (2.6)	3(0.5)	8 (1.0)		8 (5.6)	2 (2.9)	10 (4.7)		4 (12.1)	0 (0.0)	4 (10.3)	
Easy to use	12 (6.3)	13 (2.2)	25 (3.2)		25 (17.5)	6 (8.7)	31 (14.6)		7 (21.2)	0 (0.0)	7 (17.9)	
Availability	24 (12.6)	120 (20.8)	144 (18.7)		15 (10.5)	11 (15.9)	26 (12.3)		1 (3.0)	2 (33.3)	3 (7.7)	
Other reason	2 (1.1)	1 (0.2)	3 (0.4)		3 (2.1)	1 (1.4)	4 (1.9)		0 (0.0)	0 (0.0)	0 (0.0)	

*U* Urban, *R* Rural, *T* Total

### Burden of exposure to sources household of air pollution

**Fig. 3:**
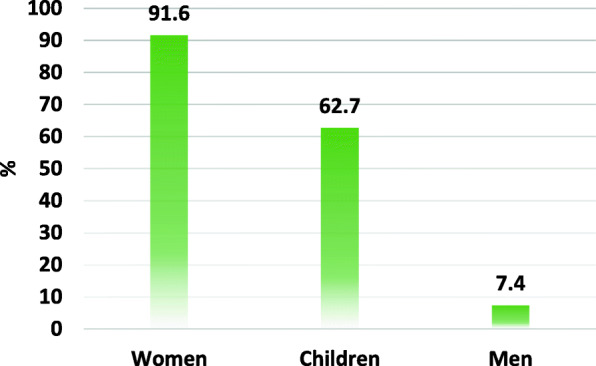
Distribution (%) of persons in charge of cooking in households of the Dschang Health District. Questionaire.

Figure 3 shows the frequency of both lone and multiple cooks in households. In 9 out of 10 households interviewed, women were reported to be the main cook. Children were involved in cooking in more than 60% of the households and men in only 7% of the households. It is to be emphasised that only the mothers in 270 (31.84%) and the children in 42(4.95%) were in charge of the cooking.

Food was cooked an average of one time (1.3 ± 0.5) per day in study households, with no variation between rural and urban areas. More than ¾ of households have been using their primary fuel type for more than five years (Table 7). Firewood has been used for the entire lifespan in almost all the households.

**Table 7:** Duration of usage of principal fuel type

Fuel types	Duration	Urban		Rural		Total		***P*** value
		***n*** (%)	(95% CI)	***n*** (%)	(95% CI)	***n*** (%)	(95% CI)	
Firewood	<= 5 yrs	14 (7.3)	(4.1–12.0)	9 (1.6)	(0.8–2.9)	23 (3.0)	(2.0–4.5)	< 0.0005
	> 5 yrs	177 (92.7)	(88.0–95.9)	569 (98.4)	(97.1–99.2)	746 (97.0)	(95.5–98.0)	
Gas	<= 5 yrs	40 (28.0)	(20.8–36.1)	10 (14.5)	(7.2–25.0)	50 (23.6)	(18.0–29.9)	0.03
	> 5 yrs	103 (72.0)	(63.9–79.1)	59 (85.5)	(75.0–92.8)	162 (76.4)	(70.1–82.0)	
Charcoal	<= 5 yrs	6 (18.2)	(7.0–35.5)	1 (16.7)	(0.4–64.1)	7 (17.5)	(7.5–33.5)	0.93
	> 5 yrs	27 (81.8)	(64.5–93.0)	5 (83.3)	(35.9–99.6)	32 (82.0)	(66.5–92.5)	
Kerosene	<= 5 yrs	4 (28.6)	(8.4–58.1)	1 (8.3)	(0.2–38.5)	5 (19.2)	(6.5–39.3)	0.19
	> 5 yrs	10 (71.4)	(41.9–91.6)	11 (91.7)	(61.5–99.8)	21 (80.8)	(60.6–93.4)	
Saw dust	<= 5 yrs	4 (15.4)	(4.4–34.9)	1 (16.7)	(0.4–64.1)	5 (15.6)	(5.3–32.8)	0.94
	> 5 yrs	22 (84.6)	(65.1–95.6)	5 (83.3)	(35.9–99.6)	27 (84.4)	(67.2–94.7)	

## Discussion

This study describes the different primary fuel types used by households in a highly populated health unit in Cameroon. These fuel types are the main sources of air pollution in households, and consequently, of many related diseases prevalent in Sub Saharan Africa. The distribution of the use of one or multiple fuel types, including the various combinations is necessary to give a real-life picture of the needs and determinants influencing the implementation of the global recommendation of adopting clean fuels for the reduction of air pollution in this area of Cameroon, in both rural and urban settings.

### Sources of household air pollution

Results of this study reveal that firewood is the main biomass fuel type used in the Dschang Health District (90%) with a significant difference between the rural and urban settings. Other fuel types including domestic gas, kerosene, charcoal and sawdust, are also used in households with slight disparities between rural and urban, yet 75% of the population strictly rely on firewood for cooking. Approximately 75% of households of the DHD use more than one source of fuel however, this proportion was very low in the rural settings (11.3%). The results of this study are in accordance with the studies conducted so far in other areas of Cameroon, showing that households mostly rely on solid fuel (especially firewood) for cooking with a higher proportion in rural areas [17–20]. Another published study conducted in another city of the same region (Bafoussam, which is the regional capital) had much lower reported solid fuel use (48%); the difference can be explained by the fact that it was conducted exclusively in an urban area with a smaller sample of household [21]. As also found in the same studies, other fuel types including domestic gas, kerosene, charcoal and sawdust are used but household access is still very limited (less than 5% at community level) for cooking; electricity is strictly used for no other purposes other than lighting. The use of clean fuels is still limited in both rural and urban areas of the DHD and therefore, the risk of developing HAP related diseases is permanently high for the people living in this area. Interventions targeting increasing population access to non-solid fuels (clean fuels) with consideration of rural and urban disparities should also be urgently implemented in this health district.

### Factors influencing the choice of fuel type

Interviewed head of households or representatives cited a number of factors influencing the choice of their cooking fuel type; affordability (59.5%) and availability (17.6%) were the main reasons. Other concerns were speed of cooking, ease of use, tradition, cleanliness and health and safety. This is in agreement with other studies carried out in different low-income areas of the world and other regions of Cameroon [17–20]. The population preferred to use wood traditionally since they found it easy to get, that is either from their farms or comparatively cheaper (pay as you go) with respect to other sources of fuel such as gas since getting gas entails disbursing large amounts of money for an initial kit (gas burner, cylinder and accessories). Given the main reasons of choice of cooking fuels presented in this study, we believe that projects aiming to support households financial access to LPG fuels can increase reduce the proportion of households relying on unclean fuels.

### To whom the burden of exposure to sources household of air pollution

This research study has shown that mothers and children were mostly those under this heavy load of HAP. Because of their customary involvement in cooking, especially women’s and children exposure is much higher than men’s as found in other studies [3, 22–25]. Some children pay the prize of either been carried on the back during cooking hours or laid to sleep on kitchen beds during the cooking process.

These women and children have been exposed to IAP almost all their lives, since almost every HH (92.7 for Urban vs 98.4 for Rural) has been using firewood as their cooking fuel for more than 5 years and only 24% of households have used LPG as their source of cooking fuel for the same duration. This is confirmed by data from the National Demographic Health Survey which presented a prevalence of 28.1% of acute respiratory infections in children under five in 2014 [26]. Showing that the continuous exposure of the population to HAP is not leaving their health indifferent. As such, studies to evaluate their effective degree of HAP exposure and interventions to aiding the population to switch from solid fuels to cleaner sources of fuel is imperative.

### Strength and limitations

This study was not without limit which however did not alter the credibility of the data presented. The principal limitation is information bias due to the fact that data collection procedures relied solely on the declaration of participants (heads of households or their representatives).

## Conclusion

This study brings out the use of wood for cooking as the main source of HAP for both urban and rural health areas of the Dschang Health District, Western Cameroon. The exclusive use of wood was greater in rural communities than in peri-urban communities. The choice of fuel type was mainly price and availability related. As such, interventions to help households (especially those who are resource poor) to adopt LPG equipment for cooking, and use in a more exclusive way is required. Education could help address some of the concerns over the use of LPG. More studies should be carried out on HAP in other regions of Cameroon so that a true picture of the nation’s state as concerns household air pollution be exposed, to bring out the relationship or association between indoor air pollution and respiratory related diseases and other health impacts, and to measure the degree of exposure to indoor air pollution.

## Data Availability

The database of this study is not available online but can be shared on request from the authors.

## Abbreviations

DHD: Dschang Health District
HA: Health Area
HH: Household
HD: Health District
IAP: Indoor Air Pollution
ARI: Acute Respiratory Infection
